# First-generation antipsychotics: not gone but forgotten

**DOI:** 10.1192/pb.bp.115.050708

**Published:** 2016-04

**Authors:** Claire R. M. Dibben, Golam M. Khandaker, Benjamin R. Underwood, Christopher O'Loughlin, Catherine Keep, Louisa Mann, Peter B. Jones

**Affiliations:** 1 Norfolk and Suffolk NHS Foundation Trust, UK; 2 University of Cambridge, UK; 3 Cambridgeshire and Peterborough NHS Foundation Trust, UK

## Abstract

**Aims and method** To identify training needs of the next generation of psychiatrists and barriers in prescribing first-generation antipsychotics (FGAs). We have surveyed psychiatry trainees in East Anglia with regard to their training experience, knowledge and attitudes to the use of oral FGAs as regular medication.

**Results** Two-thirds of trainees were aware that first- and second-generation antipsychotics (SGAs) have similar efficacy, and a similar proportion perceived the older drugs to have more or ‘stronger’ side-effects. Lack of training experience was noted as the second leading concern for prescribing FGAs. A quarter of trainees received no training exposure to the older drugs and two-thirds had never initiated these drugs themselves. Although nearly 90% of trainees felt confident about initiating an oral SGA as a regular medication, only about 40% felt confident with FGAs (*P*<0.001).

**Clinical implications** The survey highlights worrying gaps in training. FGAs can be used effectively, minimising side-effects, by careful dose titration, avoiding antipsychotic polypharmacy, high-dose, and high-potency drugs, thus ensuring they are not lost to future generations of psychiatrists.

Since the introduction of chlorpromazine in the 1950s, antipsychotics have been the mainstay of treatment for patients with schizophrenia and related psychotic disorders. Current international psychiatric practice appears dominated by the use of second-generation antipsychotic (SGA) drugs.^1-3^ The use of first-generation antipsychotics (FGAs) is perhaps often limited to rapid tranquillisation or depot injections. Healthcare professionals also report overwhelming preference for SGAs as a treatment of choice if needed for themselves.^4^ However, the rationale for this trend is not clear, especially in the face of research evidence.^5,6^ CATIE and CUtLASS 1, two large, non-commercial clinical trials comparing FGAs and SGAs for people with chronic schizophrenia in the USA and the UK respectively, have shown that the SGAs in general are no more effective (except clozapine) or better tolerated than the older drugs.^7-9^ The two classes have different side-effect profiles, yet the occurrence of extrapyramidal side-effects (EPSEs) is similar between FGAs and SGAs.^7,10^ In line with this evidence, the UK National Institute for Health and Care Excellence (NICE) guidelines do not recommend one group of antipsychotics over the other for treatment of psychosis, but suggest careful consideration of risks and benefits of each drug, taking into account patient choice.^11^

Despite the paucity of research evidence favouring one class of antipsychotics over another, the current trend in prescribing risks losing an important tool from our limited therapeutic options. It is likely that lack of training experience with FGAs will limit their use even further in the future, which is an important issue. We have carried out a survey that examines experience, knowledge and attitudes of psychiatry trainees regarding the use of oral FGAs. The survey aims to highlight training needs of the next generation of psychiatrists and identify some of the barriers in prescribing FGA medications.

## Method

We surveyed psychiatry trainees in the East Anglia region of England using an electronic questionnaire which tested their knowledge and attitudes regarding the use of oral FGAs. The following areas were included: the administrative counties of Norfolk, Suffolk and Cambridgeshire, and the unitary authority area of the city of Peterborough. Specialist training in psychiatry is organised under two National Health Service (NHS) mental health trusts: Norfolk and Suffolk NHS Foundation Trust (NSFT) and Cambridgeshire and Peterborough NHS Foundation Trust (CPFT). The survey questionnaire was initially piloted on a small group of psychiatry trainees and consultants. The survey was set up electronically via the Survey Monkey website and emailed to all trainees in NSFT and CPFT. The sample included mainly psychiatry trainees but also foundation year (FY) doctors (medical intern) and general practice (GP) trainees who were undertaking a placement in psychiatry at the time of the survey. We sent three email reminders to the trainees, and the survey was closed after 4 months. Data are presented as proportions; chi-squared test has been used to examine whether differences between groups are statistically significant.

The survey was approved by the research and development departments of both trusts as an evaluation of training.

## Results

### Response rate and sample characteristics

The survey was completed by 101 out of 146 eligible participants (69%). Response rates were similar in the two participating trusts. In total, 40% respondents were core psychiatry trainees, 40% were higher psychiatry trainees and 20% were GP trainee or FY doctors; 49% were female.

### Relative effectiveness of and concerns regarding FGAs

Whereas two-thirds (67%) of respondents noted that in terms of effectiveness FGAs were no different to SGAs (excluding clozapine), about a fifth (18%) believed that SGAs were more effective. Sixty percent of trainees had concerns about prescribing FGAs, nearly all of whom cited side-effects as their main concern. Lack of experience in using FGAs was the second most frequent concern noted by the trainees (20%). FGAs were perceived to have more side-effects or ‘stronger side-effects’ than SGAs. Trainees most commonly mentioned EPSEs, including specifically tardive dyskinesia, neuroleptic malignant syndrome, prolactin elevation, anticholinergic side-effects, QTc prolongation, cardiac toxicity and sudden death. Interestingly, criticism from colleagues was noted as a concern by one trainee.

### Training experience with FGAs

**Fig. 1:**
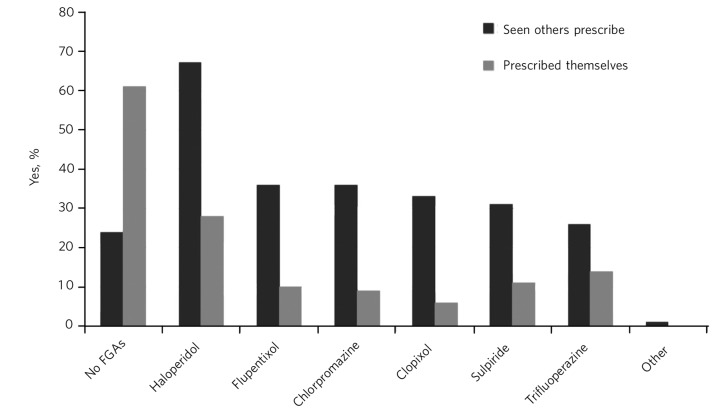
Training experience with first-generation antipsychotics (FGAs).

About a quarter (24%) of trainees said they have never seen a patient being started on an oral FGA as a regular medication excluding rapid tranquillisation or ‘as needed’ (p.r.n.) use. Haloperidol was the most commonly seen FGA, named by two-thirds of trainees. The observed use of chlorpromazine, flupentixol, sulpiride and clopixol was similar (about a third of trainees had seen each of these being used) (Fig. 1).

We asked trainees about their own prescribing practice. About 60% have never started a patient on an oral FGA as a regular medication. Among those who had, the pattern of prescribing was similar to what they had observed, namely haloperidol was the most commonly prescribed.

### Confidence regarding use of FGAs

To assess confidence in starting an oral antipsychotic as regular medication, trainees were asked to give their views on the following statements:

1. ‘I would feel confident about starting an oral FGA as a regular medication’
2. ‘I would feel confident about starting an oral SGA as a regular medication’.

**Fig. 2:**
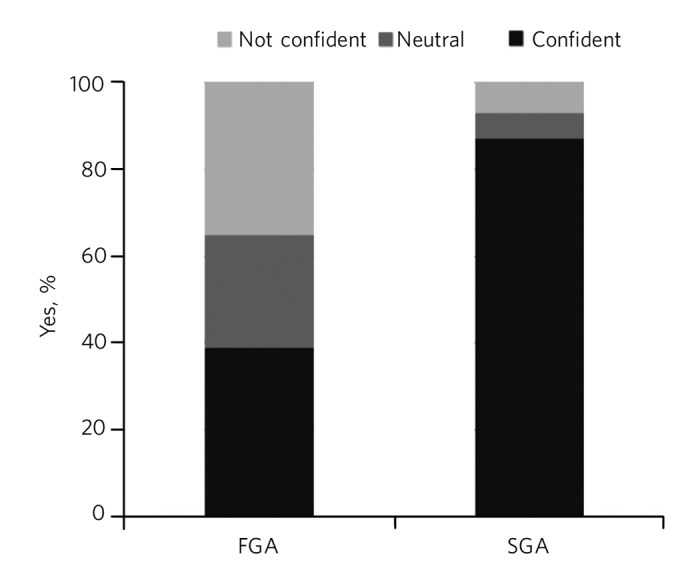
Trainee confidence regarding the use of oral first-generation antipsychotics (FGAs) and second-generation antipsychotics (SGAs).

For each statement, they could answer strongly agree, agree, neutral, disagree or strongly disagree. More than twice as many trainees felt confident about starting an oral SGA than an FGA (87% v. 39%; χ^2^ = 35.02; *P*<0.001) (Fig. 2).

## Discussion

We have surveyed 101 UK psychiatric trainees regarding their attitudes to, and experience of, prescribing different sorts of oral antipsychotic medication. Two-thirds of trainees were aware that FGAs and SGAs have similar efficacy, whereas a similar proportion were concerned about using FGAs owing to potential side-effects. Lack of training experience was noted as the second leading concern for FGA use. A quarter of trainees received no training exposure to the older drugs and two-thirds never initiated these drugs themselves. Although nearly 90% of trainees felt confident about initiating an oral SGA as a regular medication, this number was only about 40% for FGAs. These results identify current prescribing trends away from older antipsychotics and highlight worrying gaps in training.

The outcomes mirror a larger study of European psychiatry trainees which found that 93% of respondents would pick an atypical antipsychotic (SGA) for themselves.^4^ This finding was not due to ignorance of the published research, as trainees who were aware of the major studies questioning the efficacy of SGAs were even more likely to prescribe SGAs. This finding is also unlikely to be explained by length of time spent working in psychiatry as the researchers found that attitudes did not differ by length of training. Similarly, our results do not suggest that experience is necessarily a decisive factor in influencing prescribing, as the majority of trainees (76%) had experience of FGAs being used as regular medication. Experience may play some role, however, as where trainees had initiated an FGA it tended to be haloperidol, the drug they had most frequently seen being prescribed by others.

Our study goes further by examining the specific concerns which prevent FGA prescribing. Whereas side-effects were an understandable concern, particularly EPSEs, the side-effects of SGAs were felt to be less worrying. Again, this is an attitude which is not entirely supported by the evidence. In the UK CUtLASS 1 study there were no significant differences in rates of objectively assessed EPSEs between groups of patients receiving an FGA and an SGA, yet patients taking an FGA were more likely to be prescribed an anticholinergic medication.^7,10^ Similarly, the US CATIE study showed that SGAs were no better for EPSEs, negative symptoms or cognitive deficits.^9^ It is surprising that despite concerns about EPSEs, haloperidol, a potent dopamine D_2_ receptor antagonist, emerged as the most commonly used FGA in our survey. Haloperidol has a narrow therapeutic window between its antipsychotic and EPSE-inducing doses, thus carrying a higher EPSEs burden than some of the other FGAs. There is evidence that high levels of D_2_ receptor occupancy in the striatum are associated with a higher risk of EPSEs.^12^ Careful dose titration, lower total daily dosing than has been commonplace, avoiding antipsychotic polypharmacy and high-potency drugs can limit EPSEs and reduce overall side-effects in FGAs.^8,13^

Our study suggests that trainee psychiatrists lack confidence in prescribing FGAs. This is an important issue to address if the availability of these drugs is not to be lost to patients. It is possible that the results from this and other surveys reflect prescribing practice which is not evidence-based. If ignorance of the evidence is not the explanation, how might we explain this? The past decade has seen dominance of treatment by SGAs before their efficacy and side-effects were fully understood. This may have created a culture of prescribing which has not been altered by the evidence. It is possible that the relative immediacy of side-effects of potent FGAs (e.g. dystonia) compared with the more insidious effects of SGAs (e.g. metabolic syndrome) may discourage their use. Accepting the newer drugs as better and effective in treating negative and cognitive symptoms might also have thwarted drug discovery efforts for these difficult-to-treat symptoms.

The UK NICE guidelines for treatment and management of psychosis and schizophrenia in adults (published in February 2014) suggest the use of an oral antipsychotic medication in conjunction with psychological interventions for first-episode psychosis, acute exacerbation or recurrence of psychosis or schizophrenia, but do not advocate the use of SGAs as preferable over FGAs.^11^ The guidelines recommend that choice of antipsychotic medication should be made by the patient and healthcare professional together, taking into account the views of the carer if the patient agrees.^11^ Prior to the initiation of antipsychotics a full discussion of the options and relative risks and benefits of each should occur covering side-effects such as the metabolic, cardiovascular and extrapyramidal side-effects.^11^ Specifically for people with treatment-refractory schizophrenia, the guidelines recommend that clozapine should be offered to patients whose illness has not responded adequately to treatment despite the sequential use of adequate doses of at least two different antipsychotic drugs and at least one of the drugs should be a non-clozapine SGA.^11^

If the dominance of SGAs does not seem to reflect the evidence or NICE guidelines, what might be done to further explore this situation and potentially change it? One voice which is less clear in this debate is that of the patient. The results of patients surveys are mixed, some suggesting that SGAs are preferred by patients^14^ while others indicate no preference,^7^ although evidence is sparse. Therefore, it is necessary to work collaboratively with patients and carers, taking into account their views and potential risks and benefits of treatment options. Production of evidence-based patient information aimed at facilitating discussion and assisting an informed choice might be one way forward. As for increasing training exposure, consultants of today need to start considering the older antipsychotics as viable treatment options.
